# Antimicrobial stewardship of antiseptics that are pertinent to wounds: the need for a united approach

**DOI:** 10.1093/jacamr/dlab027

**Published:** 2021-03-25

**Authors:** Jean-Yves Maillard, Günter Kampf, Rose Cooper

**Affiliations:** 1 School of Pharmacy and Pharmaceutical Sciences, Cardiff University, Cardiff, Wales, UK; 2 Institute of Hygiene and Environmental Medicine, University of Greifswald, Germany; 3 School of Sport & Health Sciences, Cardiff Metropolitan University, Cardiff, Wales, UK

## Abstract

Long before the nature of infection was recognized, or the significance of biofilms in delayed healing was understood, antimicrobial agents were being used in wound care. In the last 70 years, antibiotics have provided an effective means to control wound infection, but the continued emergence of antibiotic-resistant strains and the documented antibiotic tolerance of biofilms has reduced their effectiveness. A range of wound dressings containing an antimicrobial (antibiotic or non-antibiotic compound) has been developed. Whereas standardized methods for determining the efficacy of non-antibiotic antimicrobials in bacterial suspension tests were developed in the early twentieth century, standardized ways of evaluating the efficacy of antimicrobial dressings against microbial suspensions and biofilms are not available. Resistance to non-antibiotic antimicrobials and cross-resistance with antibiotics has been reported, but consensus on breakpoints is absent and surveillance is impossible. Antimicrobial stewardship is therefore in jeopardy. This review highlights these difficulties and in particular the efficacy of current non-antibiotic antimicrobials used in dressings, their efficacy, and the challenges of translating *in vitro* efficacy data to the efficacy of dressings in patients. This review calls for a unified approach to developing standardized methods of evaluating antimicrobial dressings that will provide an improved basis for practitioners to make informed choices in wound care.

## 1. Introduction

Knowledge of wound care is derived from carvings on artefacts, ancient papyri, Sanskrit documents, religious texts, scientific works and literature. The earliest evidence found on Mesopotamian clay tablets (approximately 2500 bce) describes three stages in wound care: washing the wound, preparing topical treatments (known as ‘plasters’) and bandaging.^1^ Ancient civilizations washed wounds with beer (Sumerians), or boiled water, vinegar or wine (Greeks) and used local materials to prepare topical remedies from plants, animal products and minerals (clay and metals), whilst leaves, grasses, wool or linen acted as bandages.^2^ Consideration of wound care can be dated as far back as ancient Egypt, with the Sumerians, Greeks and Romans making significant contributions.^3^^,^^4^ The development of the chemical industry from the nineteenth century onwards began to provide antimicrobial agents that were employed in treating and preventing infection. Initially chlorine solutions were used in cleaning hospital surfaces during the 1820s and later chlorinated lime was used to disinfect obstetricians’ hands.^4^ Sodium hypochlorite was first applied to wounds by Labarraque in 1825 and formulated as EUSOL (hypochlorous acid) and Dakin’s solution (sodium hypochlorite with boric acid) in 1915. Hydrogen peroxide was discovered in 1818, but not used as an antiseptic until the late nineteenth century.^5^

Bark and pitch seeping from oil fields are two natural products that were utilized in ancient wound treatments.^2^ Fractionation of wood tar and coal tar during the nineteenth century produced many phenolic compounds that became important disinfectants and antiseptics. Creosote was used as a wound dressing by Smith in 1836 and phenol was initially used on wounds in 1860 by Küchmeister.^5^ Importantly, carbolic acid (phenol and sodium hydroxide) was applied to compound fractures by Lister in 1865, and then used to disinfect surgical instruments and operating theatres as the basis of aseptic surgery. Antiseptic solutions were widely employed in managing wounds until the end of World War II even though Alexander Fleming had demonstrated that they were rapidly inactivated by body fluids, impaired leucocyte activity and failed to permeate all areas of an irregular wound.^6^ Iodine was first used for treating wounds in France by Lugol, promoted for treating wounds by Davies in 1839 and used throughout the American Civil War. However, the painful nature of iodine, its possible influence on the thyroid function and the possibility of allergic reactions, together with observations of adverse tissue effects of traditional antiseptics in animal models,^7^^,^^8^ further limited their appeal and use declined after this time.

Since the latter half of the twentieth century antiseptic solutions that are better tolerated and have improved delivery mechanisms have been introduced into clinical practice (Table 1). These include povidone iodine (PVP-I), cadexomer iodine, chlorhexidine digluconate (CHG), octenidine dihydrochloride (OCT) and polyhexamethylene biguanide (PHMB). Although an ancient wound remedy, the use of silver in treating wounds was relatively uncommon until silver nitrate was re-introduced in 1964, closely followed by silver sulphadiazine.^9^ Honey is another ancient wound antiseptic product that lost favour in British hospitals during the 1970s, but the first modern wound care device containing medical grade honey was registered in Australia in 1999 and several types of honey are now included in formularies throughout the world.

**Table 1.** Events that have influenced the development of modern antimicrobial wound care

Intervention	Date of introduction	Location	Use
Wine, vinegar, beer	antiquity	Mesopotamia, Egypt, Greece	wound cleansing
Honey	antiquity	Mesopotamia, Egypt, Greece, India, China	in ointments applied to various wounds
Metallic silver	circa 420 bce	Persia	storage of potable water
Mercuric chloride	Middle Ages	France and Arabic civilizations	various wounds
Silver nitrate	eighteenth century	Europe	treatment of ulcers
Iodine	1829	France	various wounds
Chlorinated water and chlorinated lime	1820s	UK	hospital cleaning
	1847	Austria	antiseptic handwashing
Sodium hypochlorite	1825	France	various wounds
Creosote (wood)	1837	Ireland	dressing venereal ulcers, fistula and nasal septum
Phenol	1860	Germany	wound antiseptic
Carbolic acid	1865	UK	treatment of compound fractures
Sterile cotton/gauze	1891	USA	wound dressing
Hydrogen peroxide	1887	UK	wound antiseptic
Silver foil	1895	USA	surgical wound dressing (hernia)
Tulle gras (gauze with soft paraffin, balsam of Peru and olive oil)	1915	France	non-adherent wound dressing
EUSOL	1915	UK	wound antiseptic
Dakin’s solution	1915	UK	wound antiseptic
Chlorhexidine digluconate	1954	UK	antiseptic hand scrub and irrigating wounds
Povidone iodine	1956	USA	wound antiseptic
Cadexomer iodine	1980s	Sweden	wound dressing
Silver nitrate	1964	UK	over-granulating wounds
Silver sulfadiazine	1968	USA	infection control in burns
Polihexanide	1991	Switzerland	antiseptic solution
Octenidine dihydrochloride	1988	Germany	antiseptic solution
Medical honey	1999	Australia	topical treatment of wounds
Reactive oxygen species	2006	Belgium and UK	enzyme alginogels^a^

Here, the term antiseptic refers to a non-antibiotic antimicrobial (see section 3).

a Note that alginogels are gels rather than dressings.

The development of wound dressings was substantially influenced after the positive effect of a moist environment in promoting rapid healing was established.^10^ Occlusive and semi-permeable dressings have largely replaced dry gauze dressings and a wide range of wound dressing materials, which include paraffin gauze, polyurethanes, hydrocolloids, hydrogels, alginates and foams, have been developed since the 1980s. Integrating antimicrobial agents into these materials has provided a range of antimicrobial wound dressings.

Although the discovery of antibiotics provided an effective means to treat and prevent wound infection after World War II, the continued emergence of antibiotic resistance has compromised efficacy and the report of a pan-resistant strain of *Klebsiella pneumoniae* causing a fatal wound infection in 2016 is significant for future wound care.^11^ With decreased confidence in the effectiveness of antibiotics, the search for novel non-antibiotic antimicrobial strategies has become more important, and the need to prevent infection is more acute.

Unfortunately, bacterial resistance to antibiotics is globally increasing not only in healthcare but also in animals.^12^ It is recognized that the spread of antibiotic resistance in bacteria must be tackled in the most effective ways possible.^13^ Antibiotic stewardship combined with infection prevention comprises a collaborative, multidisciplinary approach to optimize the use of antibiotics.^14^^,^^15^ Optimizing the use of biocidal agents has also been proposed as an antimicrobial stewardship initiative to reduce risk of bacterial resistance and cross-resistance to antibiotics.^16^ As an example, reducing the use of a low concentration chlorhexidine solution (500 mg/L) for dressings on burn wounds may have increased the susceptibility of wound isolates.^17^

In addition to the antibiotics used in treating infection, effective wound management today relies on non-antibiotic antimicrobial agents employed in hand hygiene, the cleaning and decontamination of environmental surfaces and medical equipment, the decolonization of MDR strains from patients and healthcare practitioners, pre-operative skin disinfection and the appropriate use of antimicrobial dressings. However, this review is about non-antibiotic antimicrobials incorporated into wound dressings only. It aims to provide up-to-date information on their efficacy, their impact on emerging microbial tolerance and their efficacy against wound-associated microbial biofilms. This review also reflects on the appropriateness of test protocols used to measure efficacy and make a product claim. The review focuses on Europe but uses products available in the UK as examples as such products are also available in the European market.

## 2. Wounds and wound microbiology

### 2.1 Types of wound

Disrupting the normal anatomical structure and function of the skin, by either deliberate actions (such as surgery) or traumatically from chemical, physical, mechanical and thermal insults, results in a wound. The sustainable integrity of the skin is restored by a complex sequence of events that include control of infection, resolution of inflammation, removal of damaged tissue, angiogenesis, regeneration of functional extracellular tissue matrix, wound contraction, re-epithelialization, differentiation and remodelling. Wounds that complete this sequence in an orderly and timely manner are described as acute, but wounds that fail to do so are known as chronic wounds.^18^

Although non-healing wounds have been reported since the ancients Greeks, the causes of impaired healing have not been clearly established. During the last decade an insight was gained when wound chronicity was linked to the presence of microbial biofilm: light and scanning electron microscopy was used to observe biofilm in 60% of chronic wounds whereas biofilm was seen in only 6% of acute wounds.^19^ Biofilms have been detected in chronic leg ulcers,^19–21^ diabetic foot ulcers,^22^ pressure ulcers,^19^ burns,^23^ malignant wounds^24^ and surgical wounds.^25^ Recently, a systematic review and meta-analysis of published data from *in vivo* studies found the prevalence of biofilm in chronic wounds using microscopical detection methods to be 78.2%.^26^

### 2.2 Wound microbiology

Routine testing in pathology laboratories has largely relied on culture to recover potential pathogens from swabs, pus or tissue biopsies in order to determine putative identities and evaluate antibiotic susceptibilities as a guide to informed antimicrobial intervention. Standardized methodology enables international surveillance of antibiotic resistance.

Wounds often support polymicrobial communities.^27^*Staphylococcus aureus* is most frequently isolated, with *Pseudomonas aeruginosa*, *Escherichia coli*, *Enterobacter cloacae*, *Klebsiella* species, *Streptococcus* species, *Enterococcus* species and *Proteus* species also detected.^28^ Anaerobes have been underestimated;^29^ the most common species are *Peptostreptococcus*, *Prevotella*, *Porphyromonas* and *Bacteroides*, with *Finegoldia magna* and *Peptoniphilus asaccharolyticus*.^28^

In chronic wounds, culture-independent methods demonstrate the presence of more bacterial taxa than culture-dependent methods.^30–32^ Additionally, samples collected from diabetic patients treated with antibiotics in the previous 2 weeks prior to sampling had elevated abundance of *Pseudomonas* and decreased *Streptococcus* spp. compared with untreated patients,^31^ and fungal diversity increased following antibiotic administration.^32^

The distribution of microbial species in wounds is not uniform. Comparisons of bacterial abundance in chronic venous leg ulcers using qPCR showed that numbers of *S. aureus* and *P. aeruginosa* varied at different locations within the same ulcer.^33^ Next-generation DNA sequencing suggested the presence of diverse polymicrobial communities in 65 diabetic foot ulcers, but visualization with PNA-FISH and confocal laser scanning microscopy found mono-species and multi-species biofilms in the same tissue sections at locations on average 50–70 μm from the wound surface.^34^

Evidence of biofilm in wounds currently relies on scanning electron microscopy, epifluorescence microscopy or confocal laser scanning microscopy. These techniques are not yet available in pathology laboratories and there are no routine cultural methods to identify the presence of a biofilm in wounds. Clinical indicators suggestive of a biofilm in a wound are (i) failure of appropriate antibiotic therapies; (ii) recalcitrance to appropriate antimicrobial therapies; and (iii) persistent, delayed healing.^35^ As a result, a biopsy is recommended for laboratory investigation when biofilm is suspected.^36^

## 3. Application of non-antibiotic antimicrobials to wound

In this review, the term antibiotic refers to chemotherapeutic antibiotics used for topical or systemic applications. The term antimicrobial refers to both antibiotic and non-antibiotic compounds, the so-called biocidal active substances. Antiseptics refers to biocides used on intact and broken skin and on mucosa. When ‘resistance’ is mentioned in the text this often refers to antimicrobial susceptibility evaluation based on MIC determination.

### 3.1 Types of dressings and dressing functions

There are numerous dressings commercially available in the EU with varying availability throughout Europe. Table S1 (available as Supplementary data at *JAC-AMR* Online) shows dressing availability in the UK as an example. Dressings vary in their nature, composition, function, efficacy and role. The choice of the correct dressing will depend on the nature of the wound but also the healing process stage—cleansing, removal of debris, granulation, vascularization epithelialization.^37^ It is likely different types of dressing will be needed as the wound is progressing. Additional factors in choosing a dressing are patient preference and tolerance, site of the wound and cost. Ideally a dressing should ensure that a wound remain moist (under normal circumstances), free of exogenous materials (e.g. toxic chemicals, fibre materials), at the right temperature and pH, and free of infection.

Antimicrobial dressings are one type of dressing that may be used for a wound with signs of infection. They do not replace the use of systemic chemotherapeutic antibiotics if the infection spreads or becomes systemic but are used to control local wound infection. Antimicrobial dressings can be divided into those that release an antimicrobial into the wound and those that exert their antimicrobial activity following the bacterial adsorption from the wound into the dressing.^38^^,^^39^ The majority of antimicrobial dressings contain either honey or silver and their derivatives (Table S1).

### 3.2 Efficacy of biocides used in wound dressings

**Figure 1.**
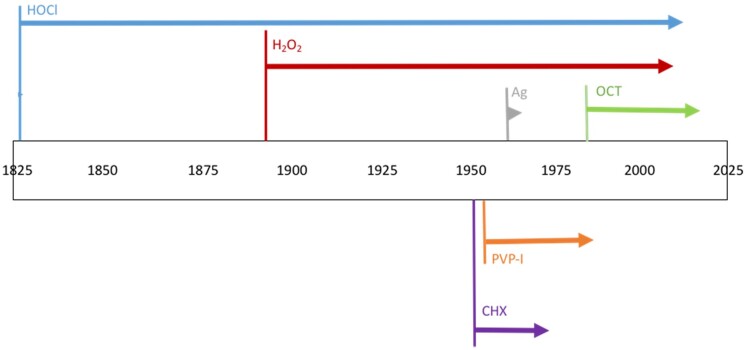
Biocide deployment and time for decreases in susceptibility to be documented. Each arrow’s length represents the time between clinical use and reported bacterial non-susceptibility.

Evidence for the antimicrobial potential of wound dressings comes from laboratory tests with either the active component alone or the entire dressing, or animal models using either explants or live animals. Clinical efficacy is determined with case studies, cohort studies or randomized controlled clinical trials. Decreased biocide susceptibility has now been described for all biocides, although evidence of bacterial decreased susceptibility may have been documented sometime after the use of a biocide in practice (Figure 1).

Epidemiological resistance is defined as an MIC above a cut-off value [where unimodal MIC or MBC/minimal fungicidal concentration (MFC) distributions were shown, epidemiological cut-offs were determined as concentrations representing ≥99.9% of the bacterial population (MIC_99.9_, MBC_99.9_ or MFC_99.9_)].^40^ An isolate is defined as clinically resistant when it is not inactivated by an in-use concentration of a biocide, or a biocide concentration that inactivates other strains of that organism, suggesting a high likelihood of therapeutic failure even when there is increased exposure.^41^ The term ‘tolerance’ describes any elevated MIC above those typical for a species.

#### CHG

**Figure 2.**
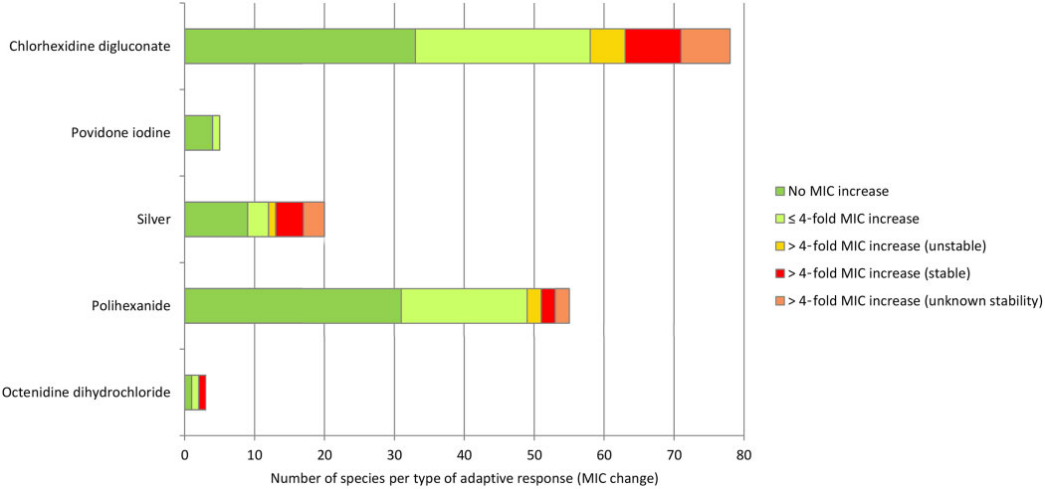
Number of species with no, ≤4-fold) or >4-fold MIC increase after low-level exposure to non-antibiotic antimicrobials used in wound dressings; (adapted from Kampf).^225^

CHG is a cationic biguanide and available as a solution for wound cleansing (e.g. at 50 mg/L) or as an impregnated wound dressing.^42^ CHG (500 mg/L; 5–15 min exposure) has been shown to be bactericidal *in vitro* against a wide range of pathogens.^43–46^ The cut-off values to determine CHG resistance proposed by Morrissey and colleagues^40^ varies between 8000 and 32000 mg/L depending on bacterial species*.* Bacterial exposure to CHG has led to >4-fold increase in MIC *in vitro* (Table 2),^47–54^ although such decreases in susceptibility may be unstable.^41^^,^^48^^,^^55^ Of note is of the possible cross-resistance to antibiotics in isolates with high CHG MIC (Table 2).^56–58^ Most isolates have so far only shown a weak or no adaptive response to CHG (Figure 2).

**Table 2.** Decreased bacterial susceptibility to biocides used in wound dressings

Examples of bacterial adaptation following exposure to biocides	Mechanisms	Cross-tolerance to antimicrobial agents	References
CHG			
>4-fold and stable MIC increase in isolates of *E. coli* (up to 500 mg/L), *K. pneumoniae* (up to 512 mg/L), *P. aeruginosa* (up to 1024 mg/L), *Serratia marcescens* (up to 2048 mg/L), *S. aureus* (up to 20 mg/L) and *Stenotrophomonas maltophilia* (up to 29 mg/L) High MIC values reported for isolates of *E. faecalis* and *K. pneumoniae* (both up to 10 000 mg/L), *P. aeruginosa* (up to 5000 mg/L), *S. aureus* (up to 2500 mg/L) and *S. marcescens* (up to 1024 mg/L)	Efflux pump encoding genes such as *qacA/B*, *qacE*, *smr* (*qacC*), on plasmids and class I integrons	Cross-tolerance possible to triclosan (*E. coli*) and hydrogen peroxide (*Acinetobacter baylyi*)	^50^
		Cross-resistance possible to ciprofloxacin, tetracycline, gentamicin, amikacin, cefepime and meropenem (*S. aureus*) and to cefotaxime, ceftazidime, imipenem, sulfamethoxazole and tetracycline (*E. cloacae*)	^56–58^
PVP-I			
No strong (>4-fold) and stable MIC increase described to date High MIC values reported for isolates of *S. aureus*, *E. coli*, *K. pneumoniae*, *P. aeruginosa* and *S. marcescens* (all up to 10 000 mg/L) *Pseudomonas cepacia* reported as a contaminant of a 10% PVP-I solution, most likely as a result of low free iodine available (0.23 to 0.46 mg/L)	No specific resistance mechanisms described to date	Cross-resistance to other antimicrobials not reported to date	^66–72^
Silver/silver nanoparticles			
>4-fold and stable MIC increase in isolates of *E. cloacae* (up to 512 mg/L), *E. coli* (up to 1024 mg/L), *K. pneumoniae* (up to 512 mg/L) and *K. oxytoca* (up to 512 mg/L); stable MIC increase in isolates with *sil* genes or efflux pumps High MIC values reported for isolates of *E. coli*, *E. cloacae* (both up to 512 000 mg/L), *P. aeruginosa* (up to 128 000 mg/L) and *K. pneumoniae* (up to 5500 mg/L)	Silver binding protein *silE* Efflux pump *silA* Membrane sensor kinase *silS* Various efflux pumps and plasmids	Cross-tolerance to copper possible via efflux pumps (*E. faecium*, *E. coli*, *Pseudomonas putida*) Cross-resistance to antibiotics possible via efflux pumps Cross-resistance to various antibiotics such as imipenem, meropenem, ceftibuten, piperacillin-tazobactam, cotrimoxazole, ciprofloxacin and gentamicin in *E. cloacae* and *E. coli*	^79^ ^,^ ^83^ ^,^ ^84^ ^,^ ^86^ ^,^ ^88–94^
Polihexanide			
>4-fold and stable MIC increase in isolates of *E. faecalis* (up to 14.5 mg/L) and *S. aureus* (up to 23.5 mg/L) No high MIC values described to date	No specific resistance mechanisms described to date	Cross-resistance to other antimicrobials not reported to date	^47^ ^,^ ^48^
OCT			
32-fold and stable MIC increase in isolates of *P. aeruginosa* (up to 128 mg/L)	No specific resistance mechanisms described so far	Cross-tolerance to CHG (*P. aeruginosa*) Cross-resistance to gentamicin, colistin, amikacin and tobramycin (*P. aeruginosa*)	^112^

The expression of efflux pumps such as the *qacA/B* gene is a well-documented mechanism resulting in elevated CHG MIC (Table 2).^59^^,^^60^ MRSA strains carrying *qacA/B* have been reported to have a CHG MIC of 256 mg/L in the presence of 3% BSA.^61^ The presence of *smr* (*qacC*), another efflux pump, was associated with a phenotypically reduced susceptibility to CHG in 88 MRSA isolates, leading to MBCs of 5, 10 and 20 mg/L in 15%, 28% and 50% of isolates, respectively.^62^ In a *Klebsiella oxytoca* isolate from a diabetic foot ulcer, the presence of *qacE* was associated with a reduced susceptibility to CHG (MIC of 30 mg/L).^63^

#### Iodophors

Iodophors (PVP-I and cadexomer iodine) facilitate the gradual release of elemental iodine when integrated into wound dressings.^64^ Typically, 10% PVP-I ointment is impregnated onto a viscose dressing and 0.9% iodine as cadexomer iodine is formulated as a paste, ointment or powder in dressings. Information on bacterial adaptation to PVP-I is limited,^65^ and data from different studies pre- and post-PVP-I exposure showed a wide MIC range in different bacterial species (Table 2).^66–72^ All isolates have so far only shown a weak or no adaptive response to PVP-I (Figure 2). Cross-tolerance to other biocides or antibiotics has not been observed.^65^^,^^73^^,^^74^

#### Silver and silver nanoparticles

Silver compounds ionize in the presence of water, bodily fluids and other exudates and antimicrobial action is dependent upon the bioavailability of the silver ion (Ag^+^).^75^ There have been many studies on the efficacy of silver ions and silver nanoparticles (AgNPs) against diverse bacterial pathogens.^76^^,^^77^ AgNPs has been reported to have a better activity than Ag^+^,^77^ and their efficacy seem to be size dependent suggesting that AgNPs with a diameter of 1–10 nm can have a direct interaction with the bacteria.^78^

The cut-off value for determining silver resistance in wound bacterial isolates varies from 27 to 512 mg/L in the literature, although resistance is often undefined or poorly evaluated.^79–82^ Bacterial exposure to Ag^+^/AgNP has led to significant changes (>16-fold) in MIC, with values reaching >1000 mg/L in *E. coli* and *E. cloacae.*^83^^,^^84^ The use of MIC as an indicator of efficacy is controversial, however, as it does not necessarily reflect the concentration of a biocide that can be attained in practice.^41^^,^^85^

Bacterial decreased susceptibility to Ag^+^/AgNP has been linked to silver resistance genes encoding for a silver binding protein (*silE*), efflux pump (*silA* and *silP*) and a membrane sensor kinase (*silS*), as well as other efflux pumps (Table 2).^79^^,^^86–93^ The effect of exposure to sublethal silver concentrations depends mainly on the presence or absence of *sil* genes.^81^^,^^84^^,^^94–97^ Upregulation of efflux pumps as well as upregulation of metal oxidoreductases has also been described as a mechanism of silver decreased susceptibility.^98^ Silver may contribute to the promotion of antibiotic resistance through co-selection, which occurs when resistance genes to both antibiotics and silver are co-located together in the same plasmid leading to the co-selection of the mobile genetic elements that they carry (Table 2).^99^ The majority of isolates have so far only shown a weak or no adaptive response to silver (Figure 2).

#### Polihexanide (PHMB)

PHMB is a cationic biguanide polymer. Preparations of PHMB are polydisperse mixtures of polymeric biguanides, with a weighted average number of 12 repeating hexamethylene biguanide units. The heterogeneity of the molecule is increased further by the presence of either amine, or cyanoguanidine or guanidine end-groups in any combination at the terminal positions of each chain.^100^

At concentrations of 200 mg/L and above, PHMB has been shown to be bactericidal (>5 log_10_ reduction in viability) within 1 h, although efficacy will decrease with lower contact time.^101–106^

Increases in MIC following PHMB exposure have been reported in a number of bacterial species.^47^^,^^48^^,^^107^^,^^108^ A stable increase in MIC has been described in *Enterococcus faecalis* (8-fold) and *S. aureus* (6-fold) but the majority of isolates have so far only shown a weak (<4-fold increase in MIC) or no adaptive response to PHMB (Figure 2).^47^^,^^48^

#### OCT

OCT is a cationic biocide and available in a gel for dressing wounds. OCT (500–1000 mg/L), often in combination with 2% phenoxyethanol, has a broad bactericidal activity in 1 min in suspension tests.^44^^,^^109–111^

Only few published data on the adaptive potential to OCT exist (Figure 2). Low-level exposure to OCT has resulted in stable 32-fold increases in MIC in *P. aeruginosa*.^112^ No specific resistance mechanisms or resistance genes associated with a reduced susceptibility to OCT have been described so far, although MFS efflux pump expression has been shown to be elevated (70-fold) in *K. pneumoniae* after low-level exposure to OCT.^113^

#### Honey

Honey is produced by honeybees foraging on blossoms and secretions from plants and insects. Being a natural product, the chemical composition of honey is variable and depends on its biological source and post-harvesting conditions. Honey destined for modern wound care products is known as medical grade honey because it is produced under hygienic conditions from relatively remote regions and is traceable and conforms to the regulatory requirements in specific countries such as Australia, Canada, USA and UK, as well as the EU. It is normally tested for antibacterial activity and contaminants, such as pesticides and antibiotics, and is incorporated into devices sterilized by gamma irradiation.^114^

Unlike antiseptics, the antibacterial properties of honey are derived from multiple factors. These include high sugar content, low water content, acidity, ability to produce hydrogen peroxide on dilution, insect-derived antimicrobial peptides, phytochemicals and methylglyoxal. Yet the relative contributions of these factors vary between different honeys.^115^ Antimicrobial components in manuka honey have not been fully characterized.^116^^,^^117^ One key inhibitor is methylglyoxal, of which levels vary for different batches of honey. Evaluating the antimicrobial efficacy of methylglyoxal from published reports may be misleading since its concentration may not be stated on wound devices or for honey samples utilized in laboratory studies. However, levels of antibacterial activity can be assured during the manufacture of devices by blending differing honey samples to achieve a specific endpoint.

The broad spectrum of antimicrobial activity of honey is well documented, with much information on manuka honey.^118^^,^^119^ Repeated subculture of bacterial suspensions in sublethal concentrations of manuka honey demonstrated that decreased susceptibility to manuka honey was transient and resistance did not arise.^120^^,^^121^

### 3.3 Antibiofilm activity

The importance and occurrence of microbial biofilms in a wound has been detailed above. The efficacy of an antimicrobial dressing should ideally be conducted against bacteria in biofilms. Most of the efficacy data of biocides relevant to dressings comes, however, from the study of planktonic bacteria. Recognizing the importance of microbial biofilms, some studies have investigated the efficacy of biocidal active substances against bacteria in biofilms and their impact on the development and mass reduction of existing biofilms.

#### CHG

There are conflicting accounts on the efficacy of CHG (500 mg/L) against single-species biofilms. While some studies showed that CHG (500 mg/L) exhibited >4 log_10_ reduction against bacteria in single species biofilms with a 5  min exposure time,^122–124^ others were unable to establish any activity (Table 3).^125^^,^^126^ The efficacy of CHG against polymicrobial biofilms seems limited.^127–131^ Biofilm maturity and bacterial species in polymicrobial communities play a role in decreasing CHG efficacy.^68^^,^^134–139^

**Table 3.** Antimicrobial efficacy of biocides used in wound dressings against biofilms

Examples of efficacy against bacteria in biofilm	Additional effect on biofilm	References
CHG		
500 mg/L CHG produced ≥4.2 log_10_ reduction in *E. coli* and *S. aureus* within 5 min, but only a 2.8–3.2 log_10_ reduction in 1 min 1000–5000 mg/L CHG resulted in ≤3 log_10_ reduction in *Burkholderia cepacia* in 1 h 20 000 mg/L CHG resulted in ≤3 log_10_ reduction in *E. faecalis* in 5 min 20000 mg/L CHG resulted in ≤3 log_10_ reduction in *E. coli* in 1 min whilst 200 mg/L 0.02% resulted in ≤3 log_10_ reduction in *E. coli* in 2 h Up to 40 000 mg/L CHG resulted in ≤3 log_10_ reduction in *K. pneumoniae* or *P. aeruginosa* in 24 h	500 mg/L CHG removed 25% biofilm mass (*Burkholderia cenocepacia*) in 15 min No removal of biofilm (*P. aeruginosa*) with 10 000 mg/L CHG in 1 h No removal of biofilm (*S. aureus*) with 10 000 mg/L CHG in 1 h	^122–126^ ^,^ ^133^
Povidone iodine/cadexomer		
1% PVP-I resulted in ≥5.0 log_10_ reduction in *S. epidermidis*, *S. haemolyticus*, *Staphylococcus simulans* or *Staphylococcus xylosus* in a single-species biofilm, even with exposure time of 30 s or 1 min 7.5% PVP-I produced ≥5 log_10_ reduction in *S. aureus* within 1 min and ≥5 log_10_ in *P. aeruginosa* within 15 min 2.5% PVP-I produced ≥5 log_10_ reduction in *S. aureus* and *P. aeruginosa* in 24 h	PVP-I able to reduce biofilm formation in *E. faecalis* and *S. aureus*	^68^ ^,^ ^135^ ^,^ ^140–144^
Silver/silver nanoparticles		
≤3 log_10_ reduction of Ag^+^/AgNP (0.01 and 25 mg/L) against *S. aureus* and mixed-species biofilms 1.0 log_10_ reduction of AgNP (total Ag concentration: 27.3 mg/L; released Ag+: 1.5 mg/L) against *P. putida*	Removal of 71% (100 mg/L NP) to 93% (25 mg/L NP) of *S. aureus* biofilm in 15 min 0% to 97% inhibition of mono species bacterial biofilms (*E. coli*, *Pseudomonas fluorescens*, *S. aureus*, *S. epidermidis, Salmonella typhimurium*) by AgNP. Biofilm protocol and concentration of AgNP account for variability in results	^76^ ^,^ ^146–152^
OCT		
1% OCT produced >6 log_10_ reduction in bacteria in biofilm in 30 min for *A. viscosus*, *P. aeruginosa* and *S. aureus* 1% OCT produced 0.6–1.8 log_10_ reduction in *E. faecalis* and *Streptococcus mutans* in mixed-species biofilms	Biofilm eradication with 0.1% OCT in 1 min (*S. aureus*) or 15 min (*P. aeruginosa*)	^110^ ^,^ ^143^ ^,^ ^157–164^
Honey		
Typical MBICs: 120 000–500 000 mg/L 5 log reduction after 24 h in *S. aureus*, *K. pneumoniae*, *P. aeruginosa*, *E. cloacae*, *A. baumannii*, *P. mirabilis* and *C. albicans*	Increased tolerance to honey, rifampicin and imipenem in clinical strain of *P. aeruginosa* isolated from a wound Bacteria produced biofilms of increased biomass compared with progenitor strains	^116^ ^,^ ^165–168^

#### PVP-I

PVP-I (1%) was shown to be efficacious (≥5.0 log_10_ reduction) in single-species biofilms, but its efficacy against mixed-species biofilms is more limited even with long exposure times (Table 3).^68^^,^^140–142^ Additional reported effect was PVP-I ability to reduce biofilm formation in *E. faecalis* and *S. aureus*.^135^ Moderate or even complete biofilm reduction by PVP-I was reported with *S. aureus* and *P. aeruginosa* (Table 3).^143^^,^^144^

#### Silver

The effect of the silver in silver-containing wound dressings against bacteria in biofilms depends on the type of dressing material and structure.^145^ Several studies reported a low efficacy of Ag^+^/AgNP against bacteria in biofilms (Table 3). ^76^^,^^146–152^ Silver alone might require a concentration of at least 0.1 mg/L to inhibit polymicrobial biofilm formation at >50% within 24 h.^153^ A comparison of seven different types of silver-coated dressing showed that there is a large variation in their ability to prevent biofilm formation of *P. aeruginosa* and *Acinetobacter baumannii* over 72 h.^154^

High biofilm biomass amount, high thickness, low surface-to-volume ratio and low roughness coefficient have been shown to compromise biocide efficacy.^147^ The combination of ionic silver with a metal chelating agent and a surfactant substantially improved the antimicrobial efficacy of ionic silver against biofilm pathogens (MRSA and *P. aeruginosa*) in a simulated wound biofilm model.^155^ Similarly, increased efficacy against *S. aureus* biofilm was reported with the combination of silver, EDTA and benzethonium chloride.^156^

#### PHMB

PHMB 0.02% and 0.04% has been shown to have low efficacy (<2 log_10_ reduction) against bacteria in biofilms.^150^

#### OCT

OCT (1000 mg/L) has been shown to produce >6 log_10_ reduction in bacteria (*Actinomyces viscosus*, *P. aeruginosa* and *S. aureus*) embedded in a biofilm, although such activity was dependent on species and whether the biofilm was polymicrobial or not (Table 3).^110^^,^^157–164^

#### Honey

Honey has been demonstrated to inhibit the formation of biofilms, as well as disrupting established biofilms of wound pathogens such as *Staphylococcus* spp., *Streptococcus pyogenes*, *P. aeruginosa*, *Proteus mirabilis*, *E. cloacae* and *A. baumannii*.^116^^,^^165–168^ These studies utilized single-species biofilms grown in microtitre plates and the range of minimum biofilm inhibitory concentrations (MBICs) recorded was 120 000–500 000 mg/L, which is less than the quantity of honey normally contained within wound dressings. However, honey is diluted by wound exudate in practice and the concentration of honey achievable within a honey-treated wound over time has not been evaluated. Bioengineered honey was found to be more effective at preventing biofilm formation than two medical grade honeys and five antimicrobial dressings.^168^

One study investigated the inhibition of wound pathogens by a manuka honey-impregnated dressing using a modified AATCC-TM100 test. Compared with control dressings without honey, >5 log_10_ reductions after 24 h were reported for *S. aureus*, *K. pneumoniae*, *P. aeruginosa*, *E. cloacae*, *A. baumannii*, *P. mirabilis* and *Candida albicans*.^169^ Another study using a chronic wound model showed that most of the commercial wound care products (only one medical grade honey) tested showed limited effects on mature biofilms.^170^

Bacterial adaptation to honey has been reported in one study, in which *P. aeruginosa* clinical isolates produced biofilms of increased biomass compared following honey exposure (Table 3).^165^

The interpretation of biocidal active substances activity against bacteria in biofilms in the wound environment is difficult to ascertain at this time. There are many biofilm models used to measure biocide efficacy (see section 4.3) and as such reported efficacy of a specific biocide varies in the literature (Table 3). Evidence—or lack of evidence—of CHG or PVP-I bactericidal efficacy against bacteria in biofilms depends on the study,^122–132^^,^^147–152^ whilst information on antibiofilm activity of PHMB is scarce.^150^ Silver and AgNP efficacy depend very much on the presence of organic materials.^145^^,^^153^^,^^154^ More information is available about honey, which was shown to have some bactericidal efficacy against bacteria in biofilms in a variety of test models, in diverse studies.^116^^,^^165–169^

### 3.4. Guidelines on using antimicrobial interventions in wound care

Non-antibiotic antimicrobial interventions play an important role in wound care. For the management of infection in diabetic foot ulcers, pressure ulcers and chronic wounds guidelines for diagnosis and treatment are available.^35^^,^^171^^,^^172^ For wound applications, the importance of balancing antimicrobial effectiveness with cytotoxicity,^173^ and the need to review an unsuccessful intervention after 2 weeks, is recognized.^174^ However, evidence of clinical efficacy is weak.^175–180^

Increased tolerance of biofilms to antimicrobials and their involvement in recurring infection has prompted the development of antibiofilm strategies. The benefits of wound debridement followed immediately by antibiotic therapy have been demonstrated^181^^,^^182^ and topical antiseptics have been suggested,^35^ despite the lack of standardized tests to evaluate antibiofilm effectiveness. Evidence of clinical efficacy of antibiofilm interventions is limited to date. Using culture-independent methodology and microscopic investigation, cadexomer iodine reduced microbial load in chronic non-healing diabetic foot ulcers containing biofilm.^183^ Similarly, the effect of duration of treatment of cadexomer iodine for diabetic foot ulcers containing biofilm on microbial load and wound healing rates were investigated.^184^ Further studies of this nature are needed to inform clinical guidance.

## 4. Measuring the activity of biocidal products/medical devices for wounds

### 4.1 Factors affecting antimicrobial efficacy

There are many factors affecting the efficacy of biocides.^41^ These have been well described for most of the active compounds found in antimicrobial dressings. Factors affecting efficacy can be separated into those depending upon the formulation/product, those depending on product usage and those depending on the target microorganisms.^41^ There are many different types of antimicrobial dressing used for a wide range of applications (Table S1). When considering antimicrobial dressings, biocides can be either an inherent part of the dressing material and not released, or the biocide diffuses from the materials into the wound, regardless of the dressing application. Either way, the available biocide concentration is paramount for activity.^85^ The impact of organic load (mainly proteinaceous in nature) in the wound or in the exudate, on antimicrobial activity, is an important factor to be considered. Additional factors contributing to a reduction of an effective concentration would be biocide adsorption to surfaces and precipitation. In the case of silver, it has been reported that the maximum attainable concentration of silver in a wound is likely to be around 1 mg/L.^185^ Above this concentration, it is expected that silver ions would complex with anions forming an ineffective insoluble silver salt.^186^ Incompatibility of the biocides with materials and excipients may also contribute to a decrease in antimicrobial efficacy. Chlorhexidine, for example, precipitates at concentrations above 0.5% w/v in the presence of inorganic acids and many salts (benzoates, bicarbonates, borates, carbonates, chlorides, citrates, iodides, nitrates, phosphates and sulphates), and incompatibilities have been reported with viscous materials such as sodium alginate, sodium carboxymethylcellulose, starch, tragacanth and hydrogel poly(2-hydroxyethyl methacrylate).^187^ Skin pH, which is usually around 5^188^ would also impact somewhat on biocidal efficacy; for example, silver efficacy will increase with alkaline pH. The pH attained in a wound is likely to be different, while microbial growth would also affect pH. Two factors of perhaps less importance are temperature and contact time. Wound temperature is unlikely to decrease dramatically (i.e. by >10°C), while dressings are usually in place for a long period of time (>24 h).

Bacterial susceptibility of different pathogens to specific biocides has been well established with most but not all biocides used in antimicrobial dressings;^41^ whilst information on silver, CHG, PHMB and PVP-I is available, information with OCT is scarce. Furthermore, a wound is likely to be polymicrobial in nature and the efficacy of a biocide will be reduced against biofilms.^41^

### 4.2 Measuring the antimicrobial activity of antimicrobial dressings

The bactericidal efficacy of biocides used in biocidal products is usually measured using defined standard efficacy tests reflecting specific applications. Until recently, in Europe, the efficacy of the biocide formulation alone was tested rather than the finished product.^189^ It is however clear that measuring the MIC of a biocide is not appropriate.^41^^,^^85^

With the many types of antimicrobial dressings available (Table S1), and the absence of specific standard tests, the main question is how the antimicrobial activity of the dressing should be measured. The efficacy of antimicrobial dressings has been tested *in vitro* during product development (Table 4), and *in vivo* using diverse animal models (Table 5).

**Table 4.** *In vitro* protocols used for testing the activity of new dressings

Antimicrobial	Protocol	Bacterial target	Reference
Chlorhexidine			
chlorhexidine	ASTM E2647-13	*A. baumannii*, *Enterobacter aerogenes*, *E. faecalis*, *E. coli*, *K. pneumoniae*, *P. aeruginosa*, *S. marcescens*, *S. aureus*	^204^
	Non-standard test		^190^
	CLSI disc diffusion	*S. aureus*	^204^
	CLSI disc diffusion	*E. coli* (ATCC 25922), *A. baumannii* (ATCC 19606), *P. aeruginosa* (ATCC 27853), *B. subtilis* (ATCC 6633), *S. aureus* (ATCC 25923), and *S. aureus* (MRSA)	^190^
	Non-standard. Immersing dressing in solution, adding bacterial inoculum for 16 h at 37°C, removing dressing and recovering bacteria from the dressing	*S. aureus* (EMRSA-15 and MSSA), *P. aeruginosa* (ATCC9027 and PA14), *K. pneumoniae* (ATCC10031), *A. baumannii* (121J6), *E. coli* (NCTC10418) and *S. epidermidis*, *C. difficile*	^198^
CHG-containing dressing	Zone of inhibition on seeded agar + dressing in broth for up to 24 h at 35°C	*S. aureus, B. subtilis, E. coli, P. aeruginosa.*	^191^
Iodine			
cadexomer iodine	Porcine *ex vivo*	*P. aeruginosa* (biofilm)	^207^
cadexomer iodine dressing	Shake flask assay: inoculum in the presence of dressing for 1–6 h at 37°C + use of neutralizer	*P. aeruginosa* ATCC 27312 and ATCC 15442, *S. aureus* ATCC 6538	^199^
cadexomer iodine	Porcine *ex vivo*	*P. aeruginosa* (biofilm)	^207^
Silver			
silver sulfadiazine		*S. aureus, P. aeruginosa*	^205^
silver sulfadiazine 1%	Non-standard *ex vivo* test on human skin	*P. aeruginosa*	^208^
silver sulfadiazine/ silver nitrate	Zone of inhibition on seeded agar	*S. aureus*	^192^
AgNPs	Zone of inhibition on seeded agar	*S. aureus* ATCC25923	^211^
silver-based dressings	Bacteria inoculated on hydrogels and recovered after 1 h at 37°C with 90% relative humidity	*E. coli* 8379, *S. aureus* 29213, *K. pneumoniae* 13883, *A. baumannii* 19606, MRSA USA300, *P. aeruginosa* PAO1 + carbapenem-resistant, *P. aeruginosa*, carbapenem-resistant *A. baumannii*	^218^
nano-composite alginate gel discs containing AgNPs	Coated discs in inoculate broth for 24 h at 37°C	*S. aureus* (ATCC 6538) and MRSA (ATCC 43300), *A. baumannii* (ATCC 19606) + 13 carbapenem- resistant strains, *E. coli* (ATCC 10536) and *P. aeruginosa* (ATCC 9027) + 1 wound isolate	^200^
200 ppm AgNPs	CLSI disc diffusion	*E. coli* (ATCC 25922), *A. baumannii* (ATCC 19606), *P. aeruginosa* (ATCC 27853), *B. subtilis* (ATCC 6633), *S. aureus* (ATCC 25923), and *S. aureus* (MRSA)	^190^
calcium alginate–nanocrystalline silver	Porcine *ex vivo*	*P. aeruginosa* (biofilm)	^207^
cotton gauze–silver sulphate	Porcine *ex vivo*	*P. aeruginosa* (biofilm)	^207^
hydrocolloid–silver	Porcine *ex vivo*	*P. aeruginosa* (biofilm)	^207^
polyacrylate–silver chloride	Porcine *ex vivo*	*P. aeruginosa* (biofilm)	^207^
silver dressings	Prevention of sedimentation biofilm formation measured by crystal violet—not quantitative—1 cm^2^ dressing added to bacterial suspension—biofilm formation measured by crystal violet	*P. aeruginosa, S. aureus, E. coli, A. baumannii*	^168^
keratin biomaterial containing AgNPs	Lysogeny broth solid plates and shake-flask method. Non-standard	*E. coli* C600, *S. aureus* RN4220, *B. subtilis* YB886	^193^
silver nanocoating	Non-standard. Immersing dressing in solution, adding bacterial inoculum for 16 h at 37°C, removing dressing and recovering bacteria from the dressing	*S. aureus* ATCC 25923 and *P. aeruginosa* ATCC27853	^206^
silver-containing crosslinked poly (acrylic acid) fibres	Zone inhibition—non-standard	MRSA USA 300	^192^
various commercially available silver dressings	Shake flask assay: inoculum in the presence of dressing for 1–6 h at 37°C + use of neutralizer	*P. aeruginosa* ATCC 27312 and ATCC 15442, *S. aureus* ATCC 6538	^201^
silver-containing dressing	Zone of inhibition on seeded agar + dressing in broth for up to 24 h at 35°C	*S. aureus, B. subtilis, E. coli, P. aeruginosa.*	^191^
antimicrobial polyur ethane foam dressing containing silver	Porcine *ex vivo* (loin roast)	*S. aureus* (DSM 20231)	^209^
commercially available silver-containing dressings	CLSI disc diffusion assay + zone of inhibition on seeded agar (some selective agar was used)	*S. aureus* (PCM 2051), *S. epidermidis* (PCM 2118), *P. aeruginosa* (ATCC 27853), *E. coli* (K12)	^194^
PHMB			
PHMB	CLSI disc diffusion	*E. coli* (ATCC 25922), *A. baumannii* (ATCC 19606), *P. aeruginosa* (ATCC 27853), *B. subtilis* (ATCC 6633), *S. aureus* (ATCC 25923) and *S. aureus* (MRSA)	^190^
cotton gauze PHMB	Porcine *ex vivo*	*P. aeruginosa* (biofilm)	^207^
PHMB	Porcine *ex vivo*	*P. aeruginosa* (biofilm)	^190^
antimicrobial gauze dressing containing polihexanide	Porcine *ex vivo* (loin roast)	*S. aureus* (DSM 20231)	^209^
OCT			
OCT	Non-standard broth dilution	*S. aureus*	^202^
	Direct contact test (according to JIS L 1902:2002)	*S. aureus*	^202^
non-antimicrobial poly urethane foam dressing intermittently irrigated with octenidine	Porcine *ex vivo* (loin roast)	*S. aureus* (DSM 20231)	^209^
Honey			
L-Mesitran Soft	Non-standard *ex vivo* test on human skin	*P. aeruginosa*	^208^
iodine, calcium alginate *Leptospermum* honey	Porcine *ex vivo*	*P. aeruginosa* (biofilm)	^207^
* Leptospermum* honey	Porcine *ex vivo*	*P. aeruginosa* (biofilm)	^207^
3 medical-grade honeys: Surgihoney RO, Activon manuka honey and Medihoney manuka honey	Prevention of sedimentation biofilm formation measured by crystal violet—not quantitative—diluted concentration of honey used	*P. aeruginosa, S. aureus, E. coli, A. baumannii*	^168^
honey-based dressings	Prevention of sedimentation biofilm formation measured by crystal violet—not quantitative—1 cm^2^ dressing added to bacterial suspension—biofilm formation measured by crystal violet	*P. aeruginosa, S. aureus, E. coli, A. baumannii*	^168^
chestnut honey- impregnated CMC hydrogel	Zone of inhibition on seeded agar	*E. coli* and *S. aureus*	^195^
honey-loaded nanofibre membrane	Non-standard broth evaluation by OD in the presence of material	*E. coli*	^201^
honey-loaded nanofibre membrane	Biofilm formation evaluated by crystal violet in presence of materials—non-standard and non-quantitative	*E. coli*	^201^
nano-composite alginate gel discs containing honey	Coated discs in inoculate broth for 24 h at 37°C	*S. aureus* (ATCC 6538) and MRSA (ATCC 43300), *A. baumannii* (ATCC 19606) + *13*, carbapenem- resistant strains, *E. coli* (ATCC 10536) and *P. aeruginosa* (ATCC 9027) + 1 wound isolate	^200^
commercially available manuka honey-containing dressings	CLSI disc diffusion assay + zone of inhibition on seeded agar	*S. aureus* (PCM 2051), *S. epidermidis* (PCM 2118), *P. aeruginosa* (ATCC 27853), *E. coli* (K12)	^194^

CMC, carboxymethyl cellulose.

**Table 5.** *In vivo* protocols used for testing the activity of new dressings

Antimicrobial	Model	Bacterial target	Study aim	Reference
Chlorhexidine				
CHG	pig	MRSA	bacterial recovery after application of CHG dressing <1.7 log_10_ cfu/g tissue after 3 days compared with 4.2 log_10_ cfu/g tissue with the placebo and 3.2 log_10_ cfu/g tissue with the gauze	^215^
	mice	—	wound healing	^198^
0.5% CHX	rat	*P. aeruginosa*	wound healing	^212^
0.5% CHX	rat	*A. baumannii*	systemic infection, and bacterial recovery	^216^
CHG/chitosan	mice	—	wound healing	^196^
Iodine				
PVI antiseptic	rat	*P. aeruginosa*	systemic infection, and bacterial recovery	^220^
PVI 3% in polyurethane foam dressing	rat	—	wound healing	^213^
cadexomer iodine	pig	*P. aeruginosa*	bacterial recovery	^207^
Silver				
silver sulfadiazine 1%	rat	*P. aeruginosa*	wound healing	^212^
silver-coated dressing	rat	*P. aeruginosa*	wound healing	^212^
calcium alginate– nanocrystalline silver	pig	*P. aeruginosa*	bacterial recovery	^207^
cotton gauze–silver sulphate	pig	*P. aeruginosa*	bacterial recovery	^207^
hydrocolloid–silver	pig	*P. aeruginosa*	bacterial recovery	^207^
polyacrylate–silver chloride	pig	*P. aeruginosa*	bacterial recovery	^207^
Acticoat^TM^	rat	*A. baumannii*	systemic infection, and bacterial recovery	^216^
silver sulfadiazine 1%	rat	*A. baumannii*	systemic infection, and bacterial recovery	^216^
silver sulfadiazine	rat	—	wound healing	^205^
silver sulfadiazine/ silver nitrate	rat	—	wound healing—skin prepared with PVI and ethanol	^192^
AgNPs	rat	*S. aureus*	bacterial recovery and wound healing	^211^
AgNPs/silver sulfadiazine	rat	—	wound healing	^200^
silver-based dressings	mice	MRSA, carbapenem-resistant *P. aeruginosa*, carbapenem-resistant *A. baumannii*	bacterial recovery and wound healing	^218^
keratin biomaterial containing AgNPs	mice	—	wound healing	^193^
polihexanide antiseptic	rat	*P. aeruginosa*	systemic infection, and bacterial recovery	^220^
OCT				
OCT	rat	*P. aeruginosa*	systemic infection, and bacterial recovery	^220^
Honey				
calcium alginate *Leptospermum* honey	pig	*P. aeruginosa*	bacterial recovery	^207^
* Leptospermum* honey	pig	*P. aeruginosa*	bacterial recovery	^207^
* Melipona scutellaris* honey	rat	MRSA ATTC43300	wound healing and bacterial recovery	^217^
chestnut honey-impregnated CMC hydrogel	mice	*—*	wound healing	^195^
Medihoney medical grade honey	rat	—	wound healing	^218^

CHX, chlorhexidine acetate; CMC, carboxymethyl cellulose.

The most common *in vitro* tests performed are based on measuring zone of inhibition of the antimicrobial dressing on seeded agar plates^190–197^ and the addition of antimicrobial dressing in an inoculated broth that can be sampled for bacterial survival over a period of time,^191^^,^^193^^,^^194^^,^^198–202^ or a combination of both (Table 4). At best, these tests provide preliminary information that the biocide can diffuse from the dressing material and show some activity against a target bacterium. The lack of a neutralization step to quench the activity of the biocide means that, at best, only a bacteriostatic activity of the biocide can be established, and as such these tests should not be used to make a claim on the efficacy of the antimicrobial dressing. Very few studies have used a standard test designed to measure the activity of an antimicrobial textile such as ASTM100:12 (Antibacterial Finishes on Textile Materials).^203^ The use of standardized tests allows a better comparison of results between studies than the use of non-standard *ad hoc* tests, which are most commonly used (Table 4).^204–206^

*Ex vivo* testing using excised animal or human skin as a substrate, or artificially damaged (e.g. puncture, burn) excised skin, provides a more accurate test protocol better representing the *in vivo* conditions of a wound.^207–210^ A number of studies have opted to use animal models: pigs, rats, mice or rabbits (Table 5). Many of these studies did not investigate the impact of bacterial infection of the wound, but the effect of the antimicrobial dressing on wound healing.^193^^,^^195^^,^^196^^,^^198^^,^^205^^,^^211–214^ A smaller number of *in vivo* studies inoculated the wound with a pathogen and investigated both bacterial survival and wound healing following the application of the dressing, providing useful information on the impact of the dressing (Table 5).^200^^,^^211^^,^^215–218^ One practical issue associated with *in vivo* protocols is the application of PVP-I or other post-operative biocides on the wound prior to the application of the antimicrobial dressing. Such practice, although ethically necessary, will impact on measuring the antimicrobial efficacy of the dressing alone. It is however apparent that even if the *in vitro* model is sophisticated enough to better represent conditions found *in vivo*, the antimicrobial dressing efficacy in patients might not be as effective.^210^

### 4.3 Measuring the antimicrobial activity of antimicrobial dressings against biofilms

If measuring the activity of antimicrobial dressings against a specific pathogen is already complex, the evaluation of their efficacy against biofilms is even more so. There are many biofilm protocols and a great divergence in opinions about their use and reproducibility. The majority of biofilm protocols use a single-species biofilm^162^^,^^170^ instead of a more complex biofilm that might represent better the polymicrobial nature of an infected chronic wound.^141^^,^^219^ Owing to the importance of the presence of a biofilm in an infected wound,^210^ a number of studies have looked at the impact of an antimicrobial dressing against the formation of biofilm rather the control of an established biofilm.^168^ These studies made use of a staining protocol that establishes biofilm biomass rather than viable bacterial count but claimed, perhaps inappropriately, antibiofilm activity of the tested dressing.^168^^,^^201^ A number of studies reported on forming single-species or complex bacterial biofilms on a substratum that was then exposed to an antimicrobial dressing for a set period of time and test conditions (temperature, pH, humidity).^141^^,^^162^^,^^170^^,^^219–221^ These protocols differ in their complexity and biofilm formation, using a range of methods such a CDC reactor,^219^^,^^221^ constant depth fermenter,^140^ colony-drip flow reactor^200^ or others.^162^^,^^170^ More advanced protocols that are trying to better mimic a wound biofilm have been reported using skin as a substratum.^207^^,^^210^ Since there are no standard tests to evaluate the efficacy of antimicrobial dressings against biofilms, the merit and relevance of each study for a particular type of wound, and their claims, need to be assessed carefully. The correlation of biofilm-based studies with the efficacy of antimicrobial dressings in practice remains to be determined.

## 5. Antimicrobial stewardship

To date limited advice on the application of the principles of antimicrobial stewardship of non-antibiotic antimicrobials pertinent to wounds is available,^222^^,^^223^ and guidance has largely centred on reducing the use of antibiotics for managing infections.^15^ One position paper^15^ recommended that only clinically infected wounds be treated with antibiotics and that infected wounds should be cultured by tissue biopsy. It proposed that short-term topical antiseptic therapy could be considered in wounds of uncertain infection status, and also as a supplement to antibiotics in infected wounds. It identified the need for clinical studies to test the efficacy of various non-antibiotic antimicrobials in treating colonized and infected wounds to determine whether antibiotic therapy could be reduced.^15^ An online course on this topic, ‘Antimicrobial Stewardship in Wound Management’, was introduced by FutureLearn in October 2019 and attracted over 8000 participants within 12 months. The potential of alternative antimicrobial strategies to minimize antibiotic usage has also been described.^224^

When applying an antimicrobial dressing to a wound, a clinical benefit should be expected. It should preferably contain an antimicrobial agent with a low adaptive response, together with the potential to prevent biofilm formation and to inhibit established polymicrobial biofilms. The duration of dressing treatment should be a short as possible and, in the case of treatment failure, it may be necessary to determine the MIC of the dominant pathogen to investigate tolerance to the non-antibiotic antimicrobial being used and direct change to another biocide.

## 6. Conclusions

Optimal management of wounds depends on avoiding the use of antimicrobial therapies when they are not indicated and prescribing appropriate antimicrobial interventions when they are indicated in order to minimize the risk of adverse effects for the patient and community. Therefore, the development of standardized methods to evaluate the effectiveness of antimicrobial dressings against both planktonic bacteria and biofilms *in vitro*, and to determine the susceptibility of microbial communities associated with wounds, would provide a stronger basis for informed choice for practitioners. However, the diversity of wound dressings and their applications, and the absence of standard tests to measure the efficacy of the antimicrobial dressing—as a product and not simply the active antimicrobial component—means that there is uncertainty as to the antimicrobial efficacy of such dressings. The use of basic *in vitro* diffusion tests relying, for example, on the size of zone of inhibition caused by the dressing is certainly not appropriate to be reported in publication. The more stringent and versatile *ex vivo* tests would provide more reliable information on the potential efficacy of the dressing to be tested *in vivo*. Overall, a better consensus on test protocols and reporting is needed to ensure claim validity and optimize non-antibiotic antimicrobial stewardship for wounds.

## Supplementary Material

dlab027_Supplementary_Data
