# Supplementary material for: Antimicrobial stewardship of antiseptics that are pertinent to wounds: the need for a united approach
Source: JAC Antimicrob Resist. 2021 Mar 25;3(1):dlab027. doi: 10.1093/jacamr/dlab027 (PMC8209993; doi:10.1093/jacamr/dlab027)
Supplement: dlab027_Supplementary_Data [file dlab027_supplementary_data.docx]

**Supplementary data**

**Table S1: Antimicrobial dressings** (https://bnf.nice.org.uk/wound-management/; accessed February 2021)

| **CHLORHEXIDINE DIGLUCONATE** | | | | | |  |
| --- | --- | --- | --- | --- | --- | --- |
| **Gauze dressing** | | Fabric of leno weave, weft and warp threads of cotton and/or viscose yarn, impregnated with ointment containing chlorhexidine acetate | | | | - Bactigras - Bactigras gauze dressing 5cm x 5cm |
| **IODOPHORES** | | | | | |  |
| **Povidone-iodine fabric dressing** | | Iodine 0.9% as cadexomer–iodine in a paste basis with gauze backing | | | | - Iodoflex paste dressing |
|  |  | Iodine 0.9% as cadexomer–iodine in an ointment basis | | | | - Iodosorb Ointment - Iodosorb ointment dressing |
|  | | Iodine 0.9% as cadexomer–iodine microbeads, 3-g sachet | | | | - Iodosorb Powder - Iodosorb powder dressing sachets |
|  | | Knitted viscose primary dressing impregnated with povidone–iodine ointment 10% | | | | - Inadine dressing 5cm x 5cm |
| **SILVER** |  | | |  | | |
| **Alginate dressings** | Calcium alginate dressing, with silver | | | - Algisite Ag - Algisite Ag dressing 5cm x 5cm | | |
|  | Calcium alginate and silver alginate dressing with polyurethane foam backing | | | - Askina Calgitrol Ag - Askina Calgitrol Ag dressing 10cm x 10cm square | | |
|  | Calcium alginate and silver alginate matrix, for use with absorptive secondary dressings | | | - Askina Calgitrol Thin - Askina Calgitrol Thin dressing 5cm x 5cm square | | |
|  | Alginate and carboxymethylcellulose dressing, with ionic silver | | | - Melgisorb Ag - Melgisorb Ag dressing 5cm x 5cm - Melgisorb Ag Cavity dressing 3cm x 44cm | | |
|  | Alginate and carboxymethylcellulose dressing impregnated with silver | | | - Silvercel - Silvercel dressing 2.5cm x 30.5cm rectangular | | |
|  | Alginate and carboxymethylcellulose dressing with film wound contact layer, impregnated with silver | | | - Silvercel Non-adherent - Silvercel Non-Adherent dressing 5cm x 5cm square | | |
|  | Calcium alginate fibre, highly absorbent, flat non-woven pads, with silver | | | - Sorbsan Silver Plus - Sorbsan Silver Plus dressing 7.5cm x 10cm | | |
|  | With silver | | | - Sorbsan Silver Ribbon - Sorbsan Silver Ribbon dressing 1g - Sorbsan Silver Surgical Packing - Sorbsan Silver Packing dressing 2g | | |
|  | Calcium alginate dressing, with silver | | | - Suprasorb A + Ag - Suprasorb A + Ag dressing 5cm x 5cm | | |
|  | Calcium alginate and carboxymethylcellulose dressing, with silver | | | - Tegaderm Alginate Ag - Tegaderm Alginate Ag dressing 5cm x 5cm | | |
|  | Alginate and carboxymethylcellulose dressing, impregnated with silver | | | - Urgosorb Silver - Urgosorb Silver dressing 5cm x 5cm - Urgosorb Silver Rope dressing 2.5cm x 30cm | | |
| **Low adherence dressing** | Three-layer antimicrobial barrier dressing consisting of a polyester core between low adherent silver-coated high density polyethylene mesh (for 3-day wear)  Acticoat | | | - Acticoat dressing 5cm x 5cm square | | |
|  | Five-layer antimicrobial barrier dressing consisting of a polyester core between low adherent silver-coated high density polyethylene mesh (for 7-day wear) | | | - Acticoat 7 - Acticoat 7 dressing 5cm x 5cm square | | |
|  | Conformable antimicrobial barrier dressing consisting of a polyester core between low adherent silver-coated high density polyethylene mesh (for 3-day wear) | | | - Acticoat Flex 3 - Acticoat Flex 3 dressing 5cm x 5cm square | | |
|  | Conformable antimicrobial barrier dressing consisting of a polyester core between low adherent silver-coated high density polyethylene mesh (for 7-day wear) | | | - Acticoat Flex 7 - Acticoat Flex 7 dressing 5cm x 5cm square | | |
|  | Non-adherent polyamide fabric impregnated with silver and neutral triglycerides | | | - Atrauman Ag - Atrauman Ag dressing 5cm x 5cm | | |
| **Foam dressings** | Three layer polyurethane dressing consisting of a silver coated layer, a foam layer, and a waterproof layer | | | - Acticoat Moisture Control - Acticoat Moisture Control dressing 5cm x 5cm square | | |
|  | Silver sulfadiazine impregnated polyurethane foam film dressing with or without adhesive border | | | - Allevyn Ag - Allevyn Ag Adhesive dressing 7.5cm x 7.5cm square - Allevyn Ag Non-Adhesive dressing 5cm x 5cm square | | |
|  | Silver impregnated polyurethane foam film dressing, with or without adhesive border | | | - Biatain Ag - Biatain Ag dressing 12.5cm x 12.5cm square | | |
|  | Silver impregnated polyurethane foam film dressing, with or without adhesive border | | | - PolyMem Silver - PolyMem Silver dressing 5cm x 7.6cm oval | | |
|  | Non-adherent, polyurethane foam film dressing with silver in wound contact layer | | | - UrgoCell Silver - UrgoCell Silver dressing 6cm x 6cm | | |
| **Soft polymer dressings** | Soft polymer wound contact dressing, with silver sulfadiazine impregnated polyurethane foam layer, with or without adhesive border | | | - Allevyn Ag Gentle - Allevyn Ag Gentle Border dressing 7.5cm x 7.5cm | | |
|  | Soft silicone wound contact dressing with polyurethane foam film backing, with silver, with or without adhesive border | | | - Mepilex Ag - Mepilex Border Ag dressing 7cm x 7.5cm | | |
|  | Non-adherent soft polymer wound contact dressing, with silver | | | - Urgotul Silver - Urgotul Silver dressing 10cm x 12cm | | |
| **Hydrocolloid dressings** | Soft non-woven pad containing hydrocolloid fibres, (silver impregnated) | | | - Aquacel Ag - Aquacel Ag dressing 4cm x 10cm rectangular | | |
|  | Non-adherent polyester fabric with hydrocolloid and silver sulfadiazine | | | - Physiotulle Ag - Physiotulle dressing 10cm x 10cm | | |
| **Silver dressings with charcoal** | Knitted fabric of activated charcoal, with one-way stretch, with silver residues, within spun-bonded nylon sleeve | | | - Actisorb Silver 220 - Actisorb Silver 220 dressing 6.5cm x 9.5cm | | |
| **PHMB (polyhexanide)** | | | | |  | |
| **Foam dressing** | | | Foam dressing with polyhexanide, without adhesive border | | - Kendall AMD - Kendall AMD Antimicrobial foam dressing 5cm x 5cm square | |
|  | | | Foam dressing with polyhexanide, without adhesive border | | - Kendall AMD Plus - Kendall AMD Antimicrobial Plus foam dressing 10cm x 10cm square | |
| **Hydrogel dressing** | | | Hydrogel containing betaine surfactant and polyhexanide | | - Prontosan Wound Gel - Prontosan Wound Gel gel dressing | |
| **Biosynthetic cellulose fibre dressing** | | | Biosynthetic cellulose fibre dressing with polyhexanide | | - Suprasorb X + PHMB - Suprasorb X + PHMB dressing 5cm x 5cm square | |
| **Low adherence dressing** | | | Low adherence absorbent perforated plastic film faced dressing with polyhexanide | | - Telfa AMD - Telfa AMD dressing 10cm x 7.5cm | |
|  | | | Low adherence dressing with adhesive border and absorbent pad, with polyhexanide | | - Telfa AMD Island - Telfa AMD Island dressing 10cm x 12.5cm | |
| **OCTENIDINE DIHYDROCHLORIDE** | | | | |  | |
| **Wound gel** | | Wound gel, hydroxyethylcellulose and propylene glycol, with octenidine hydrochloride | | | - Octenilin Wound gel - Octenilin Wound Gel dressing | |

| **HONEY** |  |  |
| --- | --- | --- |
| **Dressing** | Medical grade manuka honey | - MANUKApli Honey - MANUKApli dressing Medihoney Antibacterial Medical Honey |
|  | Honey (medical grade) 40% | - L-Mesitran SOFT ointment dressing |
|  | Medical grade, Leptospermum sp. | - Medihoney Antibacterial Medical Honey dressing - Medihoney Antibacterial Wound Gel |
|  | Medical grade, Leptospermum sp. 80% in natural waxes and oils | - Medihoney Antibacterial Wound Gel dressing |
|  | Medical grade; Bulgarian (mountain flower) 45% in basis containing polyethylene glycol | - Melladerm Plus Honey - Melladerm Plus dressing |
| **Sheet dressing** | Knitted viscose impregnated with medical grade manuka honey and manuka oil | - Actilite - Actilite gauze dressing |
|  | Knitted viscose impregnated with medical grade manuka honey | - Activon Tulle - Activon Tulle gauze dressing |
|  | Absorbent, non-adherent calcium alginate dressing impregnated with medical grade manuka honey | - Algivon - Algivon dressing |
|  | Reinforced calcium alginate dressing impregnated with medical grade manuka honey | - Algivon Plus - Algivon Plus dressing |
|  | Hydrogel, semi-permeable dressing impregnated with medical grade honey, with adhesive border | - L-Mesitran Border - L-Mesitran Border sheet 10cm x 10cm square |
|  | Hydrogel, non-adherent wound contact layer, without adhesive border | - L-Mesitran Hydro - L-Mesitran Hydro sheet |
|  | Non-adherent calcium alginate dressing, impregnated with medical grade honey | - Medihoney Antibacterial Honey Apinate - Medihoney Antibacterial Honey Apinate dressing 5cm x 5cm square |
|  | Woven fabric impregnated with medical grade manuka honey | - Medihoney Antibacterial Honey Tulle - Medihoney Tulle dressing |
|  | Sodium alginate dressing impregnated with medical grade honey | - Medihoney Gel sheet - Medihoney Gel Sheet dressing 5cm x 5cm - MelMax |
|  | Acetate wound contact layer impregnated with buckwheat honey 75% in ointment basis | - Melladerm Plus Tulle |
|  | Knitted viscose impregnated with medical grade honey (Bulgarian, mountain flower) 45% in a basis containing polyethylene glycol | - Melladerm Plus Tulle dressing 10cm x 10cm |
